# Learning from failure - rationale and design for a study about discontinuation of randomized trials (DISCO study)

**DOI:** 10.1186/1471-2288-12-131

**Published:** 2012-08-28

**Authors:** Benjamin Kasenda, Erik B von Elm, John You, Anette Blümle, Yuki Tomonaga, Ramon Saccilotto, Alain Amstutz, Theresa Bengough, Jörg Meerpohl, Mihaela Stegert, Kari A O Tikkinen, Ignacio Neumann, Alonso Carrasco-Labra, Markus Faulhaber, Sohail Mulla, Dominik Mertz, Elie A Akl, Dirk Bassler, Jason W Busse, Ignacio Ferreira-González, Francois Lamontagne, Alain Nordmann, Rachel Rosenthal, Stefan Schandelmaier, Xin Sun, Per O Vandvik, Bradley C Johnston, Martin A Walter, Bernard Burnand, Matthias Schwenkglenks, Heiner C Bucher, Gordon H Guyatt, Matthias Briel

**Affiliations:** 1 Basel Institute for Clinical Epidemiology and Biostatistics, University Hospital Basel, Hebelstrasse 10, 4031, Basel, Switzerland; 2 Cochrane Switzerland, Institute of Social and Preventive Medicine (IUMSP), Lausanne University Hospital, Lausanne, Switzerland; 3 German Cochrane Centre, Institute of Medical Biometry and Medical Informatics, University Medical Centre Freiburg, Freiburg, Germany; 4 Department of Clinical Epidemiology and Biostatistics, McMaster University, Hamilton, Ontario, Canada; 5 Department of Medicine, McMaster University, Hamilton, Ontario, Canada; 6 Institute for Social and Preventive Medicine, Zurich, Switzerland; 7 Department of Urology, Helsinki University Central Hospital and University of Helsinki, Helsinki, Finland; 8 Departments of Medicine and Family Medicine, State University of New York at Buffalo, Buffalo, NY, USA; 9 Department of Neonatology and Center for Pediatric Clinical Studies, University Children’s Hospital Tübingen, Tübingen, Germany; 10 Department of Anesthesia, McMaster University, Hamilton, Ontario, Canada; 11 Epidemiology Unit, Department of Cardiology, Vall d'Hebron Hospital and CIBER de Epidemiología y Salud Publica (CIBERESP), Barcelona, Spain; 12 Centre de Recherche Clinique Étienne-Le Bel and Department of Medicine, Université de Sherbrooke, Sherbrooke, Quebec, Canada; 13 Department of Surgery, University Hospital Basel, Basel, Switzerland; 14 Academy of Swiss Insurance Medicine, University Hospital Basel, Basel, Switzerland; 15 Center for Health Research, Kaiser Permanente Northwest, Portland, OR, USA; 16 Norwegian Knowledge Centre for the Health Services, Oslo, Norway; 17 Department of Anesthesia & Pain Medicine, The Hospital for Sick Children, Toronto, Ontario, Canada; 18 Department of Nuclear Medicine, University Hospital Bern, Bern, Switzerland

**Keywords:** Randomized controlled trial, Trial discontinuation, Slow recruitment, Ethics committees, Trial protocols

## Abstract

**Background:**

Randomized controlled trials (RCTs) may be discontinued because of apparent harm, benefit, or futility. Other RCTs are discontinued early because of insufficient recruitment. Trial discontinuation has ethical implications, because participants consent on the premise of contributing to new medical knowledge, Research Ethics Committees (RECs) spend considerable effort reviewing study protocols, and limited resources for conducting research are wasted. Currently, little is known regarding the frequency and characteristics of discontinued RCTs.

**Methods/Design:**

Our aims are, first, to determine the prevalence of RCT discontinuation for specific reasons; second, to determine whether the risk of RCT discontinuation for specific reasons differs between investigator- and industry-initiated RCTs; third, to identify risk factors for RCT discontinuation due to insufficient recruitment; fourth, to determine at what stage RCTs are discontinued; and fifth, to examine the publication history of discontinued RCTs.

We are currently assembling a multicenter cohort of RCTs based on protocols approved between 2000 and 2002/3 by 6 RECs in Switzerland, Germany, and Canada. We are extracting data on RCT characteristics and planned recruitment for all included protocols. Completion and publication status is determined using information from correspondence between investigators and RECs, publications identified through literature searches, or by contacting the investigators. We will use multivariable regression models to identify risk factors for trial discontinuation due to insufficient recruitment. We aim to include over 1000 RCTs of which an anticipated 150 will have been discontinued due to insufficient recruitment.

**Discussion:**

Our study will provide insights into the prevalence and characteristics of RCTs that were discontinued. Effective recruitment strategies and the anticipation of problems are key issues in the planning and evaluation of trials by investigators, Clinical Trial Units, RECs and funding agencies. Identification and modification of barriers to successful study completion at an early stage could help to reduce the risk of trial discontinuation, save limited resources, and enable RCTs to better meet their ethical requirements.

## Background

Randomized clinical trials (RCTs) are the optimal study design to establish the efficacy of therapeutic or preventive interventions, and are a cornerstone in drug development and comparative effectiveness research. Conducting high-quality RCTs is a challenging and resource-demanding endeavour that usually involves multiple stakeholders including clinical researchers, patients and patient interest groups, funding agencies, pharmaceutical companies, research ethics committees (RECs), and regulatory agencies.

Many unforeseen events can occur during the course of an RCT. Consequently, it is not surprising that they are often not conducted as initially planned or are prematurely discontinued.

Reasons for discontinuation of RCTs include unanticipated adverse effects (harm) [1], larger than expected benefit of an intervention (early superiority) [2], or a very low probability of detecting a designated treatment effect with continued patient recruitment or follow-up (futility) [3]. RCTs may be discontinued because the sponsor withdraws funding for strategic or administrative reasons, or because new evidence from other studies may convincingly answer the primary research question or raise serious safety issues [4]. Finally, RCTs are sometimes discontinued for practical reasons of insufficient recruitment of participants. To date the prevalence of trial discontinuation for any of these reasons cited above has not been determined. It also remains unknown whether the prevalence for specific reasons differs between trials initiated by investigators and those initiated by the industry.

### Discontinued trials due to insufficient recruitment

Difficulties in patient recruitment may necessitate amendments to the protocol. These may include prolongation of the recruitment period, broadening of inclusion criteria, addition of recruiting centres, or modifying the outcomes of interest. Some studies highlighted the high frequency of recruitment problems in RCTs (Table 1) [5-10]. However, these studies only report recruitment problems of specific trials [7,8], were based on published data [10] or the selection of trials investigated were restricted to a specific funding source [5,6]. Easterbrook et al. employed a review of study protocols [6] comparable to our approach described herein, but the data are now almost 20 years old.

**Table 1:** Examples of studies reporting about recruitment problems in randomized controlled trials (RCTs)

**Authors**	**Year**	**Data Source**	**Findings**
Charlson et al.	1984	41 RCTs (≥ 250 patients) identified by an inventory of the National Institute of Health in 1979; investigator survey was principal data source	A third of RCTs recruited fewer than 75% of their planned sample size
Easterbrook et al.	1992	720 research protocols (N = 137 RCTs) approved by REC (UK); investigator survey was principal data source	Main reason (28%) for terminating the study was slow recruitment of patients
Wilson et al.	2000	RCT that investigated two management strategies for dyspepsia in primary care (UK)	90 primary care physicians were contacted; 43 agreed to participate, 31 recruited at least one patient, only 23 recruited more than 5 patients.
Foy et al	2003	7 primary care trials of dyspepsia management in the UK	One study reached its recruitment target; five recruited less than 50% of target and three of those closed prematurely
McDonald et al.	2006	114 RCTs funded by the Medical Research Council and Health Technology Assessment (UK); full scientific applications and subsequent trial reports were principal data source	Less than a third of the trials achieved their original recruitment target
Toerien et al.	2009	133 publications of RCTs identified by a systematic literature review (restricted to six major journals)	Of those trials reporting sample size calculation, 21% failed to achieve planned numbers at randomisation and 48% at outcome assessment.

Investigators have studied patients’ attitudes to trial participation [11-13] and identified multiple barriers [14-16]. In general, patients view clinical trials as important, ethical, and as a means of attaining superior health care for future patients. However, when asked about their own participation, responders expressed more self-concern and less altruism [11]. Randomization or inclusion of a placebo arm can deter eligible patients from entering a trial [13]. Other barriers to patient participation include fear of side effects, distrust of researchers, inconvenience to everyday life, complexity of protocols, fear of deterioration of the relationship with their physician, and unawareness of trial opportunities [14,15].

In turn, attending physicians report the following barriers to an active role in trials: time constraints, lack of staff and training, worry about the impact on their relationship with patients, concern for patients, loss of professional autonomy, difficulty with the consent procedure, and lack of any reward, recognition or interest in the research question [16].

Recent research has focused on strategies of how recruitment can be improved in different settings of clinical research [17-19] and systematic reviews on the topic have identified several interventions, e.g. increasing awareness of the health problem being studied, monetary incentives, using an ‘open label’ rather than placebo design, or making trial materials culturally sensitive [20-22]. Another recent systematic review emphasized the use of qualitative methods in order to identify and overcome barriers to the recruitment activity of clinicians [23]. While trial discontinuation for apparent benefit has been investigated previously [24,25], little is known about the epidemiology and features of trials discontinued for other reasons, in particular for insufficient recruitment.

### Ethical considerations with discontinued trials

Trial discontinuation poses ethical problems. Firstly, study participants consent on the premise of contributing to the advancement of medical knowledge. The International Committee of Medical Journal Editors (ICMJE) argues that “patients who volunteer to participate in clinical trials deserve to know that their contribution to improving human health will be available to inform health-care decisions” [26]. If trials are stopped, participants should be informed about this decision and the associated reasons. However, such information may not always be given and follow-up of already recruited participants after trial discontinuation may not always be guaranteed.

Secondly, RECs face high workloads in reviewing the protocols of planned studies. However, many RECs are under-staffed and their members serve on a voluntary basis on top of their professional duties. RECs should be enabled to identify trial projects that stand a good chance of successful completion and thereby merit the investment of a thorough review by a multidisciplinary panel. According to Article 15 of the Helsinki Declaration, RECs are also entitled to monitor the progress of approved studies [27]. However, many of them may not follow up approved studies systematically despite formal requests to applicants to submit final reports or publications resulting from their research.

Thirdly, resources available for research are limited, particularly in the case of publicly funded research. Considerable waste can occur if costly RCTs need to be discontinued because assumptions about recruitment or other feasibility issues were over-optimistic [28].

Fourthly, trialists should be open about the difficulties that were encountered in failed RCTs and make their experiences available to the scientific community, in particular if the research was publicly funded. Publication of results from clinical research has been described as an “ethical imperative” [29], and in addition to data from completed studies, it has been proposed that this should also comprise information about research protocols [30]. Public access to trial protocols and publication of discontinued trials is thus of high importance to help preventing replications of unsuccessful approaches and allow the inclusion of data from discontinued trials in systematic reviews. Reports of discontinued trials are available in published literature [31-34] but remain exceptions.

A comprehensive research effort using empirical methods is necessary to better understand RCT discontinuation, to meet the associated ethical challenges, and to develop guiding principles for involved stakeholders.

## Study objectives and hypotheses

We use REC-approved RCT protocols and corresponding publications to investigate the prevalence, characteristics, and publication history of RCTs that were discontinued for different reasons, and to identify risk factors for RCT discontinuation, in particular for studies discontinued due to insufficient recruitment. The specific objectives and hypotheses are:

1. To determine the risk of RCTs to be discontinued for any reason and for specific reasons including futility, adverse events, early superiority of one intervention, and insufficient recruitment (defined for primary analysis as <90% of the planned sample size achieved, and for secondary analysis as <80%).

*Hypothesis:* The prevalence of discontinued trials among approved trials ranges from 10% to 20%; insufficient recruitment of study participants is the most frequent reason for discontinuation.

2. To determine whether the risk of trial discontinuation for specific reasons will differ for investigator- versus industry-initiated trials.

*Hypothesis:* The risk for discontinuation due to insufficient recruitment is lower for industry-initiated trials.

3. To identify characteristics of study protocols associated with premature discontinuation of RCTs due to insufficient recruitment from a list of candidate variables (Table 2). These risk factors may be modifiable or non-modifiable.

**Table 2:** Potential risk factors and protective factors for trial discontinuation due to slow recruitment

**Modifiable factors**		**Non-modifiable Factors**	
**Risk**	**Protective**	**Risk**	**Protective**
Burdensome data collection at recruiting sites	Support from a methods centre, clinical trials unit, or contract research organization	Placebo control	Active treatment as control
No professional staff at recruiting centres to manage the trial	Paid local staff at recruiting centres, dedicated central trial coordinator, patient involvement in trial planning and/or conduct	No external funding	Externally funded or fully Industry sponsored
No projection of recruitment rates	Projection of patient recruitment based on e.g. pilot trial applying the full protocol or other checks for eligible patient volume	Long duration of follow-up	Short duration of follow-up / High community interest in research topic (e.g. new technology or new treatment)
No consideration of recruitment strategies	Consideration of recruitment support strategies (e.g. regular visits/audits by PI; specific training held for recruiting staff; regular progress reports; posters and information leaflets etc.)	No research network, low trial experience	Experienced PI/steering committee/network of recruiting centres for RCTs
Single centre trial	Multicentre trial	Equivalence/non-inferiority design	Intervention only available through trial participation
Low motivation for recruiting sites	Financial incentives for recruiting staff and participants	Critically ill or paediatric patients as target population	Trial experience with certain vulnerable trial populations

Hypothesis: The more risk factors and the less protective factors are identified in a protocol, the higher the risk for discontinuation.

4. To determine the timing of discontinuation relative to the recruitment goals.

*Hypotheses:* a) Trials discontinued for futility are typically stopped at an advanced stage of the recruitment process (>60% of target sample size recruited); b) Trials exclusively discontinued due to insufficient recruitment are typically stopped at an earlier stage (<60% of target sample size recruited).

5. To examine the publication history of discontinued trials and to assess to what extent lessons learnt have been disseminated through formal publications, unpublished reports, databases or trial registers.

Hypotheses: a) Information from discontinued trials is rarely made available to others by formal publication or other forms of dissemination. b) In case of a significant result at the time of discontinuation, the results are more frequently published in a peer-reviewed journal.

## Study design and methodology

The present study addressing DISCOntinuation of RCTs (DISCO-study) is a multi-centre empirical research project that involves 4 RECs in Switzerland (Basel, Lucerne, Zurich, and Lausanne), 1 in Germany (Freiburg), and 1 in Canada (Hamilton). We have established research partnerships with each REC to access the RCT protocols approved by them between 2000 and 2003. The confidentiality of the filed study protocols is being maintained following the framework and rationale for this type of research as proposed earlier [35].

### Eligibility criteria

The DISCO-study is based on protocols of all approved clinical trials that allocated participants prospectively and concurrently to comparison groups by random or quasi-random methods of allocation (such as alternation, date of birth, or case record number) and compared one or several interventions with a placebo or sham intervention, another active intervention or no intervention. Studies comparing different doses or routes of administration of the same drug (early dose-finding studies), trials enrolling only healthy volunteers, or trials labeled as pilot or feasibility studies are included as pre-specified subgroups.

### Selection process

All study protocols approved by one of the 6 RECs between January 1^st^ 2000 to December 31^st^ 2002/3 will be screened for eligibility. For the purpose of the DISCO-study, we chose to sample protocols approved around 9 years ago to ensure that only a very small proportion of RCTs would be still ongoing at the time of our study [25].

### Definition and identification of discontinued trials

The main outcome of interest is RCT discontinuation. We define a ‘discontinued RCT’ as any RCT that was stopped before reaching at least 90% of the planned sample size due to any reason, including futility, adverse events (harm), early evidence of superiority of one intervention (benefit) and insufficient recruitment (a cut-off at 80% of the planned sample size will be considered in a sensitivity analysis). We use the following sources to identify discontinued trials:

• Internal REC reports on status or progress of approved studies,

• Correspondence between applicants and RECs with information about discontinuation,

• Any other specific method to identify discontinued trials used by the participating RECs,

• Any formal publication mentioning trial discontinuation,

• Directly contacting investigators about the status of the RCTs

### Data to be extracted

We extract data on relevant trial characteristics from protocols of eligible trials as follows:

Core protocol data

1. Centre and protocol information (e.g. local archive identification number, date of approval by REC)

2. Contact data of local and overall principal investigator (to enable contact with applicants through the local REC)

3. Trial properties (e.g. study design, number of centres, detailed information about interventions)

4. Trial funding (e.g. government, private for profit)

5. Any important changes/amendments to the protocol during the course of the trial (mainly extracted from correspondence between REC and applicant)

6. Main endpoints: Completion and publication status (e.g. trial stopped early for insufficient recruitment, trial published)

Specific protocol data

1. Clinical area (e.g. medical or surgical)

2. Setting of the trial (e.g. outpatient clinic, intensive care unit)

3. Age group of participants

4. Primary outcomes

5. Statistical analysis (e.g. planned primary analysis, intention to treat, dealing with losses to follow up)

6. Subgroups (e.g. pre-specification of subgroups)

7. Sample size, recruitment and data safety issues (e.g. planned total sample size, interim analysis, data safety monitoring board)

8. Projection of recruitment during planned enrollment time (e.g. milestones or time schedule for patient recruitment)

9. Availability of logistic/methodological support (e.g. trial support unit, structure of trial organization, paid staff at recruiting sites)

10. Strategies to support/monitor recruitment (e.g. regular newsletters, advertisement in newspapers, financial incentives)

11. Trial initiation and publication/stopping rules (e.g. industry or investigator initiated, publication constraints, sponsor rights to stop the trial)

### Data extraction process

**Figure 1:**
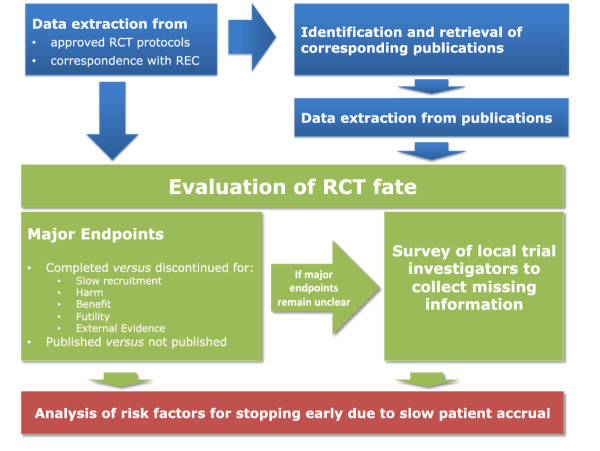
**Workflow of the DISCO study.** RCT, randomized controlled trial; REC, research ethics committee.

We use a web-based password-protected database (Squiekero, http://www.squiekero.org) for data extraction. A manual with definitions and rules for data extraction for each variable has been compiled, updated and shared among all staff involved in data extraction at the 6 study sites. About 15 methods-trained investigators extract data from trial protocols. The course of action is illustrated in Figure 1 and listed in Table 3.

**Table 3:** Steps for identification of discontinued trials and data extraction; REC, regional ethics committee

**Steps**	**Actions**
1	Identification of protocols of RCTs submitted 2000 to 2002 with the help of REC staff members
2	Extraction of trial characteristics from eligible protocols and attempt to clarify completion of trials through filed correspondence between the REC and applicants
3	Electronic search for publications (e.g. MEDLINE, EMBASE, Google Scholar) of eligible trials using filed information such as key words from protocol title/intervention or names of investigators
4	REC in charge will contact the applicants using a standardized questionnaire to ask about reasons of discontinuation and the availability of any formal publications, unpublished reports or other information from eligible trials (only in case trial completion and publication status remain unknown after searching filed correspondence and comprehensive publication search)
5	The REC in charge may send several reminders or contact applicants by phone if necessary
6	After receiving responses from applicants the data collection process will be finalized
7	The analysis database will contain only anonymous data with trial identification numbers

We conduct calibration exercises in which extracted data from several protocols will be compared and thoroughly discussed in order to ensure consistency between the investigators. This process is crucial given that some of the variables to be extracted require personal judgement. We plan to extract 30% of eligible protocols independently and in duplicate and conduct random checks for consistency in remaining protocols.

### Search for publications

If no information about the publication status of a trial is given in the REC files, we conduct electronic searches in literature databases including Medline, Embase, Google Scholar, Cochrane CENTRAL register of clinical trials, CINAHL, AMED, and topic specific databases. We also search trial registers such as ClincialTrials.gov, ISRCTN, the WHO International Clinical Trials Registry Platform and registers of sponsors, if publicly available. We use key words from the protocol title and interventions, study acronyms, and names of the investigators as search terms. Depending on the database, we limit the searches to randomized trials in humans and take into account possible time of publication. If potential publications are found, we attempt to identify the main publication of the trial by retrieving the full text. We also check whether the main publication refers to other publications of the trial (especially rationale and design papers). From the included publications, 2 investigators extract data independently and in duplicate on the following topics: author and publication information, trial properties, study funding, clinical area, methodological quality, enrolment and follow-up, outcomes, analysis, subgroups, and sample size/recruitment.

## Risk factor analysis for discontinuation due to insufficient recruitment

In a sub-study, we will compare trials that were discontinued due to insufficient recruitment with completed trials. From this subgroup, we will exclude trials that (i) used cluster randomization (because they differ from trials that randomize individuals in issues of recruitment), (ii) are still ongoing in 2012, and (iii) have unclear completion status or reasons for discontinuation other than insufficient recruitment. Trials that were discontinued due to insufficient recruitment will be considered as “cases” and all other completed trials as “controls”.

## Data management and statistical analysis

Data management and database cleaning will be carried out using R version 2.15.1 (The R project for statistical computing, http://www.r-project.org). We will read the definitive dataset into STATA (version 12.1, STATA Corporation, Austin/Texas, USA) for statistical analyses. The reasons for trial discontinuation will be analysed using descriptive statistics, including risks (cumulative incidences) of discontinuation expressed as percentage with 95% confidence intervals. In the sub-study on trial discontinuation due to insufficient recruitment, potential risk factors (hypothesis 3) will be analysed using multivariate hierarchical logistic regression models with protocol-level variables as fixed effects and the ‘participating centers’ (i.e. the RECs) as a random effect. This approach will account for variability from two sources, i.e. within and between centers. To minimize the risk of overfitting and data-driven associations, we have pre-specified risk factors and confounding variables for the statistical model and limited their number to obtain no less than 10 events (i.e. discontinued trials) per explanatory variable in the resulting multivariable logistic regression models [36].

Risk factors will include: Placebo/no treatment control *versus* active intervention, single center *versus* multicenter trial, no or inadequate *versus* adequate projection of recruitment during planned enrolment period, and absence *versus* presence of methodological/logistical support. Potential confounders will include: presence *versus* absence of industry funding/involvement, parallel *versus* cross-over/factorial trial, and the planned total number of participants.

We will calculate odds ratios with 95% confidence intervals. Statistical test results with two-sided P < .05 will be regarded as significant. We expect that the proportion of missing data for the above specified variables will be low because the information to be collected from a trial protocol is either very basic or it is about the presence or absence of information in the protocol (e.g. pilot trial mentioned or not). Further, we will contact site investigators for clarifications/missing information if necessary. In our primary analysis, we will only consider protocols with complete data (complete cases analysis). In a second step, missing data will be imputed using multiple imputation techniques; based on this imputed dataset, we will conduct a sensitivity analysis (all case analysis). Furthermore, we will conduct bootstrapping for internal model validation.

## Estimated sample size

In a previous study, protocols of randomized drug trials submitted between 1989 and 1998 were analysed [37]. Fifty-seven of 531 trials (11%) were discontinued for different reasons. In 22 cases (39%) the reason was insufficient recruitment of participants. In the cohort of trials established in Freiburg (Germany), 74 of 299 studies submitted in 2000 (25%) were discontinued [38]. Taking into account these results and the available literature [39,40] we estimate that about 10% to 20% of trials started are discontinued due to insufficient recruitment. Based on information by the collaborating RECs and published data, we anticipate that we will identify over 1000 eligible RCT protocols approved by the participating RECs between 2000 and 2002/3 and that about 15% of these RCTs were discontinued due to insufficient recruitment. Under the assumption of a minimal odds ratio to be detected of 2.0 and 150 of 1000 RCTs to be stopped due to insufficient recruitment, we calculated the power to detect such an association between an exposure factor (e.g. single centre status) and the binary outcome of discontinuation due to insufficient recruitment. As an example, the power to detect an association for an exposure factor is 88% if the prevalence of this factor in the “control trials” is 20% (Table 4). Therefore a sample size of 1000 protocols should be sufficient for our planned analyses.

**Table 4:** Power calculations for different prevalences of a single risk factor for trial discontinuation; RCT, randomized controlled trial; OR, odds ratio

**Prevalence (%) of risk factor**		
**Completed RCTs**	**RCTs discontinued due to slow accrual**	**Study power (%) to detect OR = 2.0**
10	18	65
20	33	88
30	46	95
40	57	96

## Discussion

The DISCO study will determine the prevalence of RCTs discontinued for a variety of reasons, differences between industry and investigator-initiated RCTs, risk factors for discontinuation due to insufficient recruitment from RCT protocols, the stage at which RCTs are discontinued, and examine the publication history of completed and discontinued RCTs. To achieve these goals a cohort of over 1000 RCTs in various medical fields will be established based on the protocols approved at participating RECs over a four-year time period. Through this publication we intend to make our study objectives and methods transparent [41].

### Strengths and limitations of the protocol

In this empirical study we use robust methodology including a transparent and systematic process to identify eligible RCTs, to extract relevant characteristics from protocols, and to search for corresponding publications. The collaboration with 6 RECs in 3 different countries should enhance the generalizability of our results. Approximately 1000 RCTs will provide sufficient statistical power for the planned analyses and likely represent one of the largest cohorts in the field of empirical trial research.

The rigor of our study depends not only on the level of detail and quality of protocols, but also on the completeness of the correspondence and amendments between the investigator and the REC. We will systematically search these files to capture any relevant information about the course of the trial, as well as on issues of recruitment or changes in design or modification of primary endpoints. In case we are not able to evaluate the completion or publication status of the trial based on the filed documents at the local REC, applicants or principal investigators will be contacted through local RECs. Experience from one of our previous projects suggests that most applicants will respond [38].

### Beyond discontinued trials

The DISCO-study offers the possibility to investigate discrepancies between protocols and subsequent publications e.g. with regard to pre-specified and reported primary endpoints, statistical analyses, or sample size. As an example, judging the credibility of subgroup effects when reading trial publications is challenging and, following recent recommendations, it is crucial to pre-specify anticipated subgroup effects before the analysis [42]. The DISCO-study will allow investigations about the planning and reporting of subgroup analyses in RCTs from various medical fields.

### Comparison with similar studies and protocols

The STEPS study was an epidemiological survey of 114 RCTs funded by the UK Medical Research Council and Health Technology Assessment (HTA) Programme [28]. Less than one-third of included trials recruited their original target number of patients within the time originally planned. Trials that reached their originally specified sample size more frequently had a dedicated trial manager, were cancer or drug trials, or offered treatments to patients exclusively available within the trial. The most commonly reported strategies to improve recruitment were newsletters and Email reminders, but the investigators could not determine whether these measures were causally linked to changes in recruitment [28].

In contrast to the STEPS study, our database will consist of RCTs that were not funded by a single agency but funded by various sponsors and sources including the industry, public, and in-house sources of university-affiliated hospitals. We will determine if the risk factors identified in the STEPS study can be reproduced within our more diverse and much larger trial cohort.

The recruitment performance of local sites within a multicentre trial is the key to successful trial completion. Recently, Dal-Ré et al. proposed the disclosure of recruitment performance of local sites within multicentre trials in publicly available trial registries [43]. The rationale is that this would render the trial recruitment process more transparent and trialists more accountable, because their recruitment performance could be followed by patient organizations, sponsors, and the scientific community. The DISCO-study captures the recruitment goals of the local site and the total across all study sites, which will allow further insights into these important planning issues.

The recently finished IMPACT-study by Oude et al. (personal communication), investigated barriers and facilitators for successful patient recruitment to gynecology/obstetrics trials in the Netherlands [44]. The group established a nationwide cohort of trials with recruiting physicians being interviewed about crucial determinants of recruitment at a center level. Furthermore, using a nested case–control design, they interviewed patients who refused or consented to participate in order to identify factors associated with their decision. In a second cohort study, the group investigated the association between successful recruitment and issues such as hospital organization and design of trials prospectively registered in the Netherlands Trial Register. This study, especially the latter part, has goals similar to ours. However, the methods and study population to identify risk factors are different. In IMPACT, data about potential risk factors were gathered through a questionnaire while we use data from approved protocols; and we focus exclusively on RCTs whereas IMPACT included non-randomized studies as well. The IMPACT investigators also outlined a problem regarding generalizability of potentially identified risk factors for insufficient recruitment which also applies to our protocol: on a patient level, participation or non-participation in a clinical trial might predominantly depend on characteristics of a trial and its target population; therefore overall predictors for insufficient recruitment may not be identified. We may consider this issue in sensitivity analyses e.g. through stratification by medical field. However, full data collection will demonstrate the number of events of interest; this will limit the number of variables that can be investigated in multivariable logistic regression models.

### Implications and significance

The DISCO-study will provide important insights into the prevalence and features of RCTs that were discontinued for different reasons. RCTs are highly resource demanding endeavours with stakeholders including patients, clinicians, investigators, funding agencies, and industry. Effective recruitment strategies and the anticipation of problems are key issues in the planning and evaluation of trials by investigators, Clinical Trial Units, RECs and funding agencies. With the identification of potential barriers to successful study completion, the DISCO-study will help reduce the risk of premature trial discontinuation and save limited research resources. Furthermore, as outlined in the Ottawa Statement [30], RCTs imply ethical obligations to research participants. When consenting to a trial, participants accept the potential of harm that may occur to them. Their risk of harm is primarily counterbalanced by the presumed overall social good resulting from the advancement of medical knowledge. We anticipate that evidence from the DISCO-study will underpin the current efforts to enhance the transparency, standardisation and accessibility of trial information. Such improvements are crucially needed to meet the ethical obligations of RCTs and to prevent that a decline in numbers of volunteering participants will ultimately make clinical research impossible.

## Competing interests

This project is supported by the Swiss National Science Foundation (grant 320030_133540/1) and the German Research Foundation (grant EL 544/1-2). JWB is funded by a new investigator award from the Canadian Institutes of Health Research and the Canadian Chiropractic Research Foundation. KAOT is supported by unrestricted grants from the Finnish Cultural Foundation. The funding sources have no role in the design and conduct of this study and the writing of this manuscript.

## Authors’ contributions

EE, MB, and BK have designed the study and written the manuscript. They are also involved in data collection. JY, YT, AB, TB coordinate data extraction from protocols, extract data and have revised the manuscript. RS developed the web-tool for data extractions. AA, JM, MS, KAOT, IN, AL, MF, SM, and DM are involved in data extraction from protocols and have revised the manuscript. EA, DB, JWB, IG, FL, AN, RR, SS, XS, PV, BJ, MS, and MW extract data from publications and have revised the manuscript. BB, HB, and GG supported the initiation of the study, provided logistical support, and revised the manuscript. All authors approved the final version before submission.

## Ethical approval

The participating Research Ethics Committees approved the study or explicitly stated that no ethical approval was necessary.

## Pre-publication history

The pre-publication history for this paper can be accessed here:

http://www.biomedcentral.com/1471-2288/12/131/prepub
